# Antibiotic Disruption of the Gut Microbiota Enhances the Murine Hepatic Dysfunction Associated With a High-Salt Diet

**DOI:** 10.3389/fphar.2022.829686

**Published:** 2022-02-11

**Authors:** Zheng Zhang, Mengjie Li, Bo Cui, Xiao Chen

**Affiliations:** ^1^ State Key Laboratory of Biobased Material and Green Papermaking, School of Food Science and Engineering, Qilu University of Technology, Shandong Academy of Sciences, Jinan, China; ^2^ College of Health Sciences, Shandong University of Traditional Chinese Medicine, Jinan, China

**Keywords:** high-salt diet, antibiotic exposure, gut microbiome, mitochondrial function, hepatic steatosis

## Abstract

Epidemiological and experimental evidence indicates that antibiotic exposure is related to metabolic malfunctions, such as obesity and non-alcoholic fatty liver disease (NAFLD). Liver impairment and hypertrophy of adipose cells are related to high salt consumption. This research aims to investigated the physiological mechanism of a high salt diet (HSD) enhanced antibiotic-induced hepatic injury and mitochondrial abnormalities in mice. The mice were fed a HSD with or without penicillin G (PEN) for 8 weeks and the gut metabolome, untargeted faecal metabolomics, and intestinal function were evaluated. The results revealed that HSD, PEN and their combination (HSPEN) significantly changed the gut microbial community. HSPEN mice exhibited more opportunistic pathogens (such as *Klebsiella* and *Morganella*) and reduced probiotic species (including *Bifidobacterium* and *Lactobacillus*). The main variations in the faecal metabolites of the HSPEN group were identified, including those connected with entero-hepatic circulation (including bile acids), tryptophan metabolism (i.e., indole derivatives) and lipid metabolism (e.g., erucic acid). Furthermore, increased intestinal permeability and immunologic response caused greater hepatic damage in the HSPEN group compared to the other groups. These findings may have important implications for public health.

## Introduction

Non-alcoholic fatty liver disease (NAFLD) is a chronic reversible disease of the liver characterised by metabolic syndromes such as hepatic manifestations (Rinella, 2015; Younossi et al., 2016). NAFLD normally begins with fatty deposits in the liver, followed by liver damage, including steatohepatitis, inflammation, fibrosis, cirrhosis, and hepatocellular carcinoma (Syn et al., 2009; De Medeiros and Lima, 2015). NAFLD patients do not exhibit secondary accumulation of hepatic fat due to alcoholism, hepatitis B infection, hepatitis C infection, the use of steatogenic medication, or hereditary disorders (Bellentani et al., 2010; Chalasani et al., 2012). Over the last decade, there has been increasing research on NAFLD, providing a better understanding of this disease. This research has indicated that NAFLD is related to obesity, hyperlipidaemia and gut microbial dysbiosis (Bellentani et al., 2010; Chen et al., 2020b).

Trillions of microorganisms occupy the human body and play a role in the health of the body (Huttenhower et al., 2012). The gut contains the densest habitat of microorganisms, with a microorganism biomass of approximately 0.15 kg (Sender et al., 2016). The physiology, metabolism, immunity, and overall health of the host are significantly influenced by the microbial community. Individual genetics and environmental factors, especially diet, affect the structure of the intestinal microbiota (David et al., 2014; Rothschild et al., 2018). For instance, individuals who eat non-Western and/or fibre-rich diets have an abundance of the *Prevotella* genus (De Filippo et al., 2011; Smith et al., 2013), since *Prevotella* hydrolases are specifically designed to degrade plant fibres (Purushe et al., 2010). Consistent with the association between *Bacteroides* and diets rich in animal proteins and saturated fats, the majority of *Bacteroides*-specific carbohydrate-active enzymes (CAZymes) (50%) are dedicated to animal carbohydrates (Costea et al., 2017). Our recent studies found that a high-salt diet (HSD) changed the structure of the intestinal microbiota, further causing health problems such as liver steatosis, hypertension and constipation (Zhang et al., 2019b; Chen et al., 2020a). Additionally, previous studies have confirmed that a HSD is closely related to hypertrophy of adipose cells (Dobrian et al., 2003), NAFLD (Lanaspa et al., 2018) and hepatic fibrosis (Wang et al., 2016). Given that the colon is the primary organ responsible for sodium homeostasis, excessive salt intake can disturb the intestinal microbiota and the “gut-liver” axis (Lienhard et al., 2012). Specifically, microbial alterations impair the metabolome in the gut. Several toxic metabolites, such as deoxycholic acid and endogenous alcohol, enter the liver *via* the hepatic portal vein and contribute to liver steatosis and mitochondrial abnormalities (Bourzac, 2014; Yuan et al., 2019). Mitochondrial dysfunction is one of the pathogenic mechanisms of NAFLD (Sumida et al., 2013). Mitochondrial dysfunction plays a crucial role in the course of hepatic steatosis in patients and animal models, not only influencing liver lipid homeostasis but also leading to accumulation of reactive oxygen species (ROS), which gives rise to lipid hyperoxidation, cytokine overproduction and hepatocyte death (Begriche et al., 2013; Koliaki et al., 2015). Additionally, environmental risk factors, including antibiotic use (Mahana et al., 2016) and heavy metals (Kim and Lee, 2014), may promote mitochondrial dysfunction in the liver and contribute to the development and progression of NAFLD.

Antibiotics have been utilised extensively for decades. However, the presence of these substances in the environment has only become of concern more recently. Antibiotics can be excreted in the faeces and urine, and given that it is a common practice to use animal faeces as a fertilizer in many countries, the underlying effect of residual antibiotics on the environment and crops has garnered growing international attention (Sarmah et al., 2006). The effects of antibiotics on the intestinal microbiota has attracted increasing concern, with shifts in dominating flora, reduced community diversity (Grazul et al., 2016), proliferation of drug-resistant or opportunistic bacteria (Buelow et al., 2017), and even delayed colonization by beneficial bacteria after drug administration (Hertz et al., 2020) observed. Furthermore, a troubling consequence of antibiotic therapy is the persistence of antibiotic-resistant genes in the human intestine (Jernberg et al., 2010). Therefore, antibiotics can have a long-term effect on the intestinal microbiota and its metabolites, even affecting liver function through the “gut-liver” axis (Mahana et al., 2016). However, to the best of our knowledge, there is no published research on the role of the liver and gut microbiota in the molecular response to the combined influences of antibiotic exposure and HSD.

To this end, this study examined the liver function of mice exposed to long-term low-dose penicillin G followed by administration of a HSD, and compared the liver function of these mice to that of control mice. It was assumed that the gut microbiota is an essential enabling factor in NAFLD, and both antibiotic treatment and HSD are able to change the gut microbiota and hepatic mitochondrial function. In order to develop novel intervention strategies, a more comprehensive understanding of the contribution of the microbiome to NAFLD pathogenesis is necessary.

## Materials and Methods

### Animals and Exposures

Thirty-two male C57BL/6J mice (8 weeks old; specific pathogen-free) with production license number SCXK (Lu) 2019-0003 were obtained from Pengyue Laboratory Animal Technology Co., Ltd. (Jinan, China). Every mouse was caged individually in a germ-free cage under a ventilated 12 h light/dark cycle; the environmental ambient temperature was 25°C and the working humidity was 60–70%. The Animals Ethics Committee of the Experimental Animal Centre of Shandong University of Traditional Chinese Medicine (No. SYXKLU20170022, Jinan, China) approved this study, and the study was conducted in accordance with EU Directive 2010/63/EU for the care and use of laboratory animals.

**FIGURE 1:**
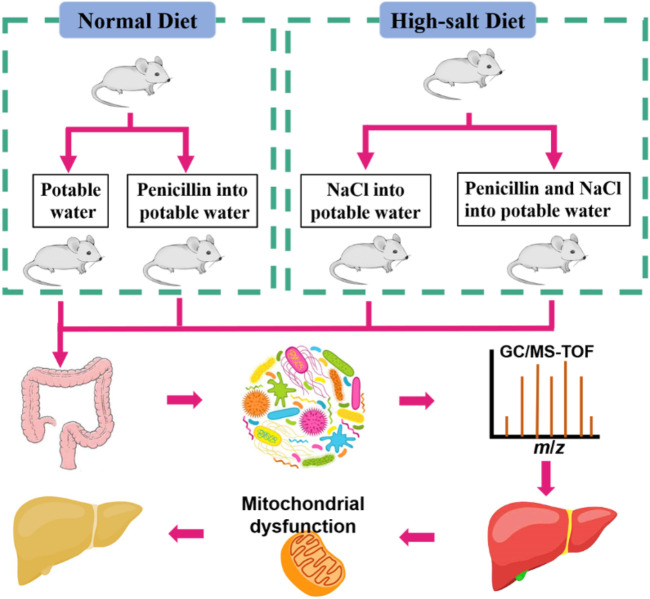
Experimental process.

During the first week, animals were fed a commercial laboratory diet and water *ad libitum* to acclimatise them to the surroundings, followed by the experimental period for 8 weeks. The animals were randomly assigned into four groups, each containing eight mice: ND (normal diet, control), HSD (supplement with 4% NaCl), PEN (6.8 mg/L Penicillin G added to the drinking water dams) (Mahana et al., 2016), and HSPEN (HSD plus PEN) (Figure 1). 1% NaCl was supplemented in the potable water of the HSD and HSPEN mice (Wilck et al., 2017). Gamma-irradiated (25 kGy) diets were obtained from BiotechHD Co., Ltd (Beijing, China) and formulated according to the American Institute of Nutrition (AIN)-93G purified diet criterion to meet the nutritional needs of the growing mice. The food was kept at −20°C and was replaced daily. The weights of the mice were recorded weekly during the 8 weeks of the study.

### Sample Preparation

At the completion of the treatment protocol, the mice were moved to single disinfected cages. Faeces were collected per the protocol described in our previous research (Chen et al., 2020a). After 12 h of fasting, the animals were anaesthetised by injection of 1% sodium pentobarbital (40 mg/kg; Sigma Chemical Co., St Louis, MO, United States) through the abdominal cavity. Blood was acquired from the eye socket of each mouse and then celiotomy was performed. Next, hepatic and colonic samples were collected. The blood specimens were centrifuged at 1000 g for 30 min at 25°C, and the serum was collected.

### Levels of Serum Biomarkers, Liver Injury, and Intestinal Dysfunction

Using an automatic biochemical analyser (Au680, Beckman Coulter, Inc., Brea, CA, United States), the following biochemical indicators in serum were evaluated: glucose (GLC), glutamic-pyruvic transaminase (ALT), glutamic-oxalacetic transaminase (AST), alkaline phosphatase (ALP) and triglycerides (TG). Serum interleukin-17a (IL-17a) and IL-22 levels were determined using ELISA kits (Gersion, Beijing, China) per the manufacturer’s instructions. TG and malondialdehyde (MDA) in the hepatic tissues were measured according to previously described methods (Chen et al., 2020a).

The serum levels of D-lactate (D-LA) and diamine oxidase (DAO) were measured by specific ELISA kits (Gersion), according to the manufacturer’s instructions, to estimate the intestinal permeability. Colon tissues were opened longitudinally, and the colonic contents were scraped and gathered to measure the pH value (Zhang et al., 2019a). The tissues were fixed in formalin immediately and then embedded in paraffin and sectioned. Routine haematoxylin-eosin (H&E) staining was then performed. The hepatic tissues were stained using Oil Red O (ORO) for 15 min. Then, the tissues were rinsed and counterstained with haematoxylin for 5 min to observe the accumulation of lipids in the liver.

The expressions of CD4^+^ and CD8^+^ lymphocytes and IgA (1:100, OriGene Technologies, Inc., Rockville, MD, United States) in the colonic mucosal epithelium of the mice were detected by immunohistochemistry (IHC) using monoclonal antibodies of CD4^+^, CD8^+^ and IgA. Image Pro-Plus 6.0 software (Media Cybernetics, lnc., Rockville, MD, United States) was used to analyse and measure the positive control cells (tan−yellow cytoplasm) of CD4^+^, CD8^+^ and IgA lymphocytes in the colon. The protein expression levels were calculated according to the protocol of Mao et al. (2014).

### Mitochondrial Function and Integrity in the Liver

The collagenase perfusion technique was applied to isolated hepatocytes from mice, as previously described (Spach et al., 1991). Afterwards, the hepatocytes were washed with chloromethyl-X-rosamine (Mito Tracker; Thermo Fisher Scientific, Rockford, IL, United States) for 30 min, fixed with 4% paraformaldehyde in PBS for 15 min, and then washed with PBS three times. Then, 4′,6-diamidino-2-phenylindole (DAPI; Thermo Fisher Scientific) was employed to stain the hepatocytes for 5 min. The fluorescence in separate liver cells was observed with a Nikon AR1 confocal microscope (Nikon Corporation, Tokyo, Japan).

A transmission electron microscope (TEM; Tecnai G2 Spirit, FEI, United States) equipped with an Eagle camera (FEI) was employed to evaluate the ultrastructure of the hepatocyte mitochondria. In brief, the hepatic tissues were fixed in 2.5% glutaraldehyde at 4°C for 2 h, post-fixed in 1% osmium tetroxide at 4°C for 1 h, dehydrated, and then embedded in epoxy resin. After slicing, ultrathin hepatic tissue sections (70 nm) were placed in copper grids, double-stained with 8% uranyl acetate and lead citrate for comparison, and detected with the TEM system.

Mitochondria were separated from the hepatic tissues by way of traditional differential centrifugation, as previously described (Chweih et al., 2015). Respiratory substrates, including 300 μmol ethylene glycol bis(β-aminoethyl ether)-N,N′-tetraacetic acid (EGTA), 10 μmol Amplex Red, and 1 U/mL horseradish peroxidase with 2.5 mmol malate plus 5 mmol pyruvate were blended with 0.5 mg/ml of the mitochondrial suspension. The mitochondrial H_2_O_2_ release rate was assessed using a RF-5301PC spectrofluorometer (Shimadzu, Japan), as previously described (Navarro et al., 2017).

### Quantitative (q)RT-PCR

For the qRT-PCR, total RNA was isolated from the liver tissue using a RNAiso Plus kit (Tiangen Biotech Co. Ltd., Beijing, China) according to the manufacturer’s instruction. Quantitation of extracted RNA was confirmed by absorbance measurements at 260 nm and evaluation of purity was performed by the ratio of OD260/280 nm above 1.80. The RNA (25 ng) was collected and reverse-transcribed into cDNA using a cDNA Synthesis Kit (Tiangen Biotech) according to the manufacturer’s instruction. All the RT-PCR steps were performed using a 25 μl scale in a 7500 Fast Real-Time PCR System (Applied Biosystems, United States) in triplicate using SYBR Green I dye assays (Tiangen Biotech) for *Mfn1*, *Drp1*, and *Fis1* in the liver. Relative quantification was quantitated using the ΔΔCt method, normalizing the housekeeping gene expression to *Actb* (β-actin; Gene ID: 11461). Primers used were as previously described (Chen et al., 2014) and follows: *Mfn1* (F: ATT​GGG​GAG​GTG​CTG​TCT​C; R: TTC​GGT​CAT​AAG​GTA​GGC​TTT), *Drp1* (F: CGG​TTC​CCT​AAA​CTT​CAC​GA; R: GCA​CCA​TTT​CAT​TTG​TCA​CG), *Fis1* (F: AAG​TAT​GTG​CGA​GGG​CTG​TT; R: GGC​AGA​GAG​CAG​GTG​AGG), and *Actb* (F: GGA​TGC​AGA​AGG​AGA​TCA​CTG; R: AGA​TCC​ACA​CGG​AGT​ACT​TG).

### Gut Microbiota Profiling

A commercial DNA extraction kit (Qiagen, GmbH, Hilden, Germany) was used to extract total genomic DNA from the faecal samples per the manufacturer’s instructions. The procedures for the separation of DNA, Illumina MiSeq amplicon sequencing, and library generation were consistent with the general methods previously described (Zhang et al., 2020). In order to analyse the diversity of bacteria, universal primers (515F: 5′-GTGCCAGCMGCCGCGG-3′ and 907R: 5′-CCGTCAATTCMTTTGAGTTT-3′) and HiFi Hot Start Ready Mix (KAPA Biosystems, Woburn, MA, United States) were used to amplify the V4−V5 variable regions of the 16S rRNA gene. Gel electrophoresis was used to observe amplicon quality, purification was performed with AMPure XP beads (Agencourt), and PCR was used for secondary amplification. After purification again with the AMPure XP beads, a Qubit dsDNA assay kit was used to quantify the final amplicon. Equal amounts of purified amplicon were collected for subsequent sequencing.

The original sequencing data were in FASTQ format. Paired-end reads were pre-treated using Trimmomatic software (Bolger et al., 2014) to test and remove undefined bases, and then matched by FLASH software (Reyon et al., 2012) following the parameters as previously reported (Chen et al., 2020a). Sequence data were processed using the Quantitative Insights into Microbial Ecology (QIIME, version 1.8.0) pipeline, with 75% of the bases having quality scores above 20 (base-calling accuracy of 99%) (Caporaso et al., 2010). All effective tags were clustered into operational taxonomic units (OTUs) at a 97% stringency threshold using the workflow provided by the QIIME package (Edgar, 2013). The RDP classifier (Wang et al., 2007) (confidence threshold was 70%) was employed to annotate and blast all typical reads against the Greengenes (16S rDNA). Blast was used to annotate and blast all representative reads against the Unite database (ITSs rDNA) (Altschul, 1990).

### Faecal Metabolomic Profiling

In brief, 20 μl of L-2-chlorophenylalanine solution (1 mg/ml in distilled water) was used as an internal standard. The faecal pellets were added and the metabolites were isolated with chloroform and methanol. After homogenization and ultrasonic treatment, the supernate was transferred to a fresh glass vial. An Agilent 7890A GC system (Agilent Technologies, Santa Clara, CA, United States) equipped with a Pegasus HT time-of-flight mass spectrometer (Leco, Saint Joseph, MI, United States) was employed to analyse the microbial-host co-metabolites, according to the parameters previously described (Zhang et al., 2019b).

### Data Analysis

All results are presented as the mean ± standard deviation (SD) of the repeated tests or as the median with interquartile range. Independent measurements were compared with *t*-tests using SPSS version 22.0 software (SPSS Inc., Chicago, IL, United States) as appropriate. One-way analysis of variance was used to analyse the differences between groups; multiple groups were compared with the Turkey post-hoc test. Significance was determined at *p* value < 0.05.

## Results

### Characteristics of the Liver in the Established Model

**FIGURE 2:**
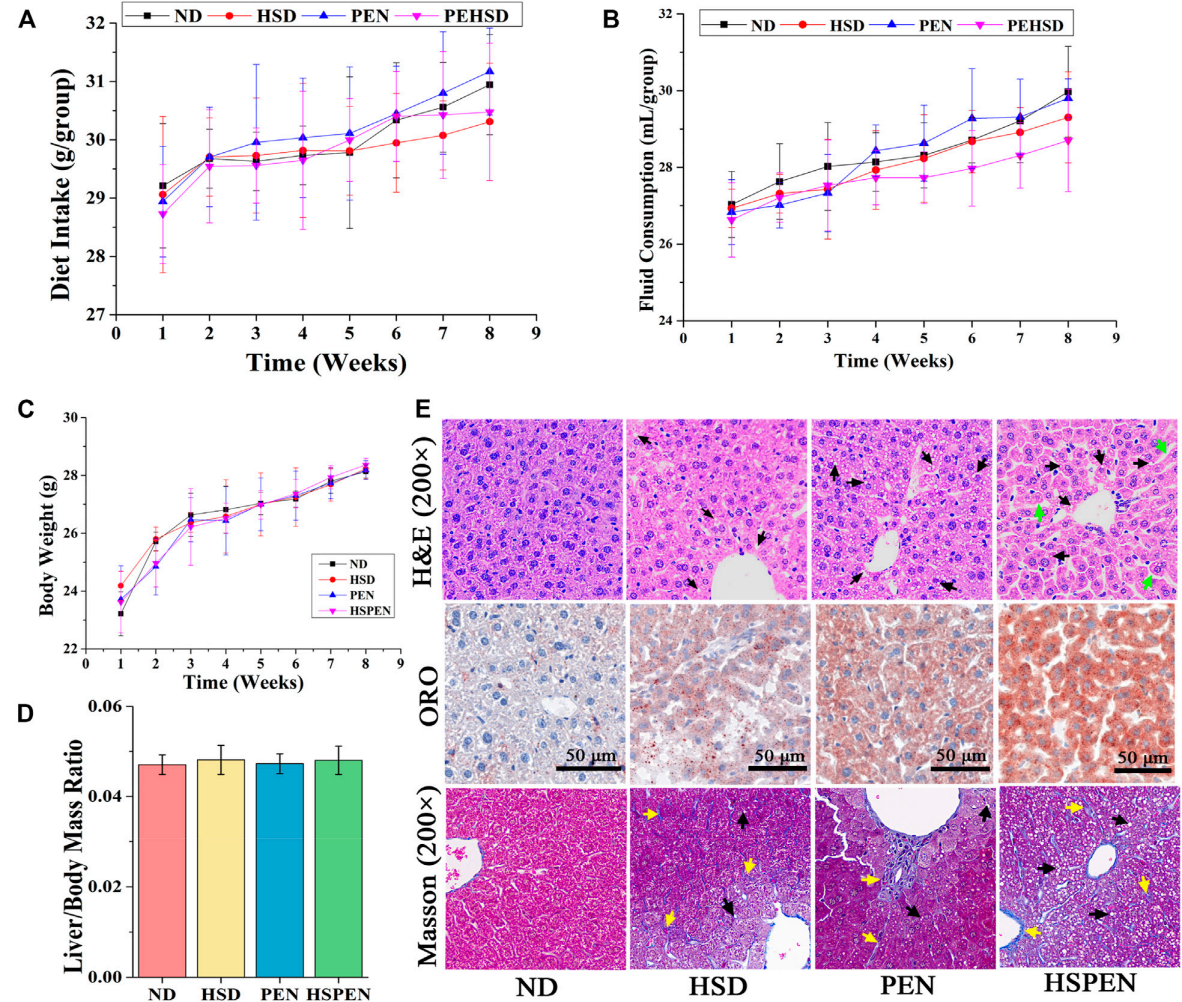
High-salt diet, Penicillin G, and their combination caused liver injury in mice. The daily diet intake **(A)**, fluid consumption **(B)**, and body weights **(C)** of the different groups throughout the experiment. **(D)** Liver/body mass ratio. Values are presented as the mean ± SD; *n* = 8. **(E)** Representative mouse liver sections with H&E, ORO and Masson staining. Black arrows indicate ballooning degeneration in liver tissues. Green arrows indicate loss of cellular boundaries around the central vein of hepatocytes. Yellow arrows indicate obvious fibrosis in liver tissues.

After 2 months of feeding, the effects of high salt intake and antibiotic exposure on the liver tissues were evaluated according to the clinical biomarkers in serum, the biochemical parameters in liver tissues, and the liver photomicrographs. The ALT, AST, ALP, GLC, and TG contents in serum in the HSPEN group were obviously increased compared to those in the ND group (*p* < 0.05; Table 1). In particular, the serum contents of AST, GLC, IL-17a and IL-22 and the concentrations of TG and MDA in the liver tissues of the HSPEN-treated mice were dramatically elevated compared to the other three groups (*p* < 0.05; Table 1). Furthermore, throughout the experiment, there were no significant differences in diet intake (Figure 2A), fluid consumption (Figure 2B), body weight (Figure 2C), and liver/body mass ratio (Figure 2D) among the groups of animals (*p* > 0.05).

**TABLE 1:** Levels of clinical biomarkers in the serum and liver tissues of the different groups.

Sample	Name	ND	HSD	PEN	HSPEN
Serum	ALT (IU/L)	53.34 ± 3.49^a^	62.69 ± 11.76^ab^	65.32 ± 11.62^b^	80.94 ± 7.40^b^
	AST (IU/L)	138.47 ± 32.74^a^	185.90 ± 51.92^b^	207.66 ± 26.85^b^	259.80 ± 34.04^c^
	ALP (U/L)	69.00 ± 5.42^a^	88.75 ± 14.99^b^	74.38 ± 7.58^a^	93.60 ± 8.08^b^
	GLC (mmol/L)	7.81 ± 1.44^a^	16.65 ± 3.49^b^	17.57 ± 3.11^b^	23.47 ± 2.32^c^
	TG (mmol/L)	0.62 ± 0.05^a^	0.75 ± 0.19^ab^	0.91 ± 0.21^bc^	1.11 ± 0.33^c^
	IL-17a (pg/ml)	24.64 ± 2.71^a^	28.67 ± 1.90^b^	41.81 ± 1.93^c^	49.19 ± 5.72^d^
	IL-22 (pg/ml)	17.03 ± 1.15^a^	19.65 ± 2.33^ab^	21.06 ± 0.86^b^	26.67 ± 1.53^c^
Liver	TG (mmol/gprot)	0.15 ± 0.03^a^	0.23 ± 0.04^b^	0.32 ± 0.06^c^	0.47 ± 0.09^d^
	MDA (mmol/mgprot)	1.34 ± 0.27^a^	1.76 ± 0.51^b^	1.66 ± 0.44^b^	2.01 ± 0.72^c^

Data are expressed as the mean ± SD; *n* = 8. Horizontally, ^a–d^Values followed by different letters are significantly different (*p* < 0.05).

H&E, ORO and Masson staining were employed to examine the micromorphology of the liver tissues (Figure 2E). In the ND group, the liver structure was normal, with the nucleus located in the centre of the hepatic cells; there was no apoptosis, and hepatocytes were evenly distributed and arranged in a rope-like pattern. In the other three groups, the liver cords were loose and disorderly; there was neutrophilic granulocyte infiltration in the hepatocytes, increased lipid accumulation (black arrows), loss of cellular boundaries around the central vein of the hepatocytes (green arrows), and obvious fibrosis in the liver tissue (yellow arrows), particularly in the HSPEN group. This suggests the presence of liver histological changes (Figure 2E). These results indicate that hepatic steatosis and liver injury had developed in the HSD- and PEN-treated mice.

### Effects of HSD and Antibiotic Exposure on Mitochondrial Function in the Liver

**FIGURE 3:**
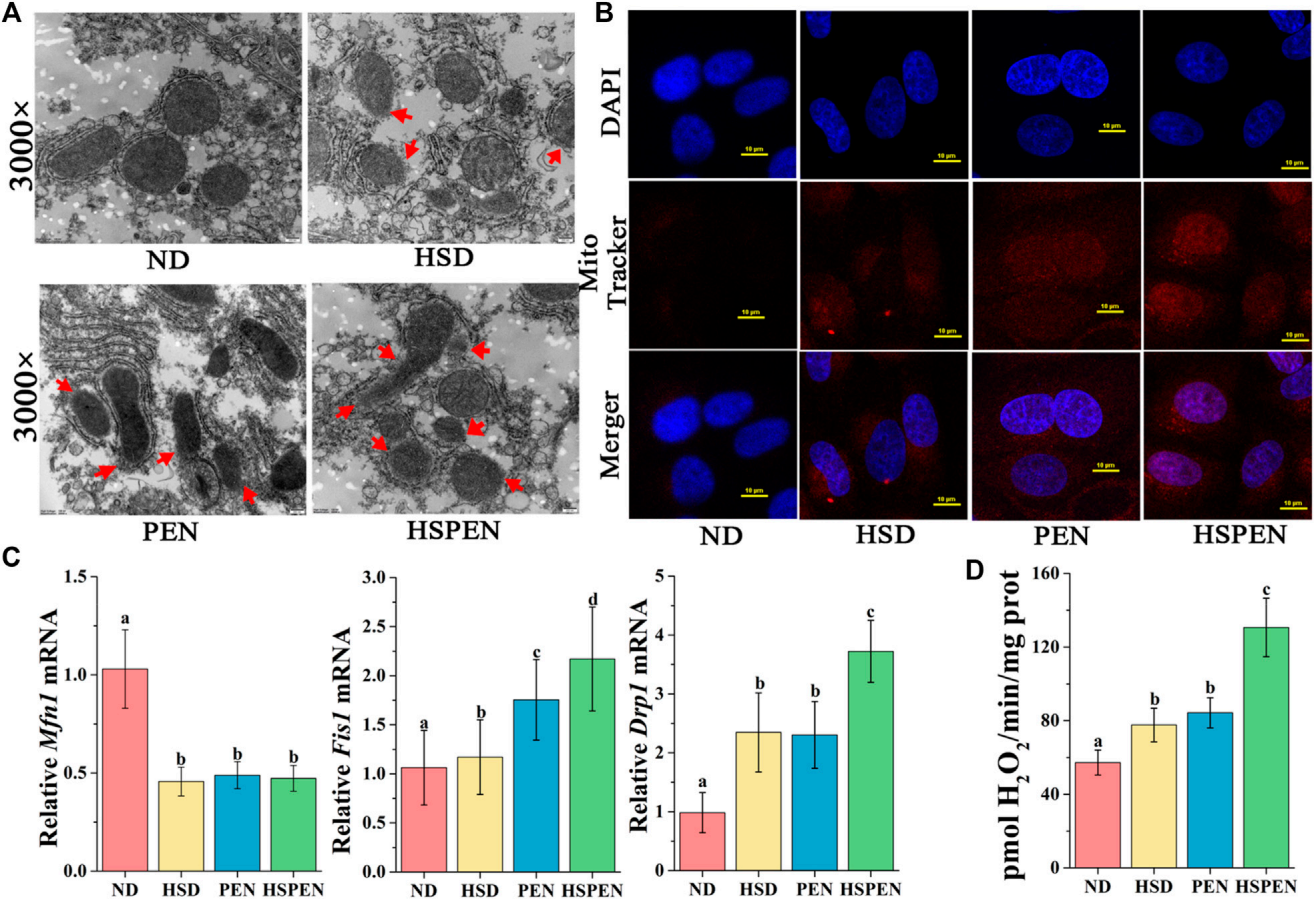
HSD and antibiotic exposure induce mitochondrial dysfunction in the liver. **(A)** Swollen mitochondria and mitochondrial membrane rupture (red arrows) in the livers of mice as shown in typical TEM photomicrographs (×3000 magnification). **(B)** MitoTracker red was used to stain hepatocytes for 30 min and liver cells were analysed using confocal microscopy (scale bar: 10 μm). **(C)** The mRNA levels of the mitochondrial fusion marker *Mfn1* and fission markers *Drp1* and *Fis1* in the liver tissue, normalized to levels in the liver tissue of the ND group. **(D)** Mitochondrial release of H_2_O_2_ in mouse liver. Data are shown as the mean ± SD; *n* = 8. Different letters were significantly different (*p* < 0.05).

Although the mechanisms underlying the progression of hepatic steatosis are not fully understood, accumulating evidences have indicated that mitochondrial dysfunction plays a dominant role in the development of steatohepatitis (Koliaki et al., 2015; Zhang et al., 2021). In the present study, the molecular mechanisms of steatosis induced by high salt intake and antibiotic exposure were explored by assessing mitochondrial integrity and function in mouse livers (Figure 3). The TEM results revealed mitochondria swelling and disruption of the mitochondrial membrane (red arrows) in the hepatocytes of mice treated with HSD and penicillin, both individually and in combination (Figure 3A). Since mitochondria are the main producers of ROS in cells, mitochondrial ROS outputs in liver cells were assayed. DAPI-localized nuclei and Mito Tracker Red-localized mitochondria were separately observed with confocal microscopy according to the blue and red fluorescence (Figure 3B). The results indicated that the mitochondrial ROS levels in the HSPEN group were higher than those in the ND, HSD, and PEN groups. Given the detrimental effects of high salt intake and antibiotic exposure on mitochondrial integrity in steatohepatitis, we examined whether HSD, penicillin and the combination of the two could change the expression of genes regulating mitochondrial integrity. A family of mitochondrial guanosine triphosphatases, including Mfn1, Drp1, and Fis1, regulate mitochondrial metabolic function (Romanello and Sandri, 2016). Mfn1, situated in the outer membrane of mitochondria, is a mitochondrial fusion protein that can accelerate mitochondrial fusion (Santel and Fuller, 2001). Drp1 and Fis1 can promote the division of mitochondria (Smirnova et al., 2001; Koch et al., 2005). In comparison with the ND group, the *Mfn1* mRNA level was reduced and the *Drp1* and *Fis1* mRNA levels were significantly increased in the HSD, PEN, and HSPEN groups (*p* < 0.05). Moreover, the *Drp1* and *Fis1* mRNA levels were significantly elevated in the HSPEN group compared to the HSD and PEN groups (*p* < 0.05; Figure 3C). Furthermore, the production of mitochondrial H_2_O_2_ was dramatically increased in HSPEN-treated mice compared with ND-, HSD- and PEN-treated mice (*p* < 0.05; Figure 3D). These findings suggest that the HSD- and PEN-treated mice exhibited dysfunction and abnormalities of mitochondria.

### HSD and Antibiotic Exposure Induce Enteric Dysbacteriosis in Mice

**FIGURE 4:**
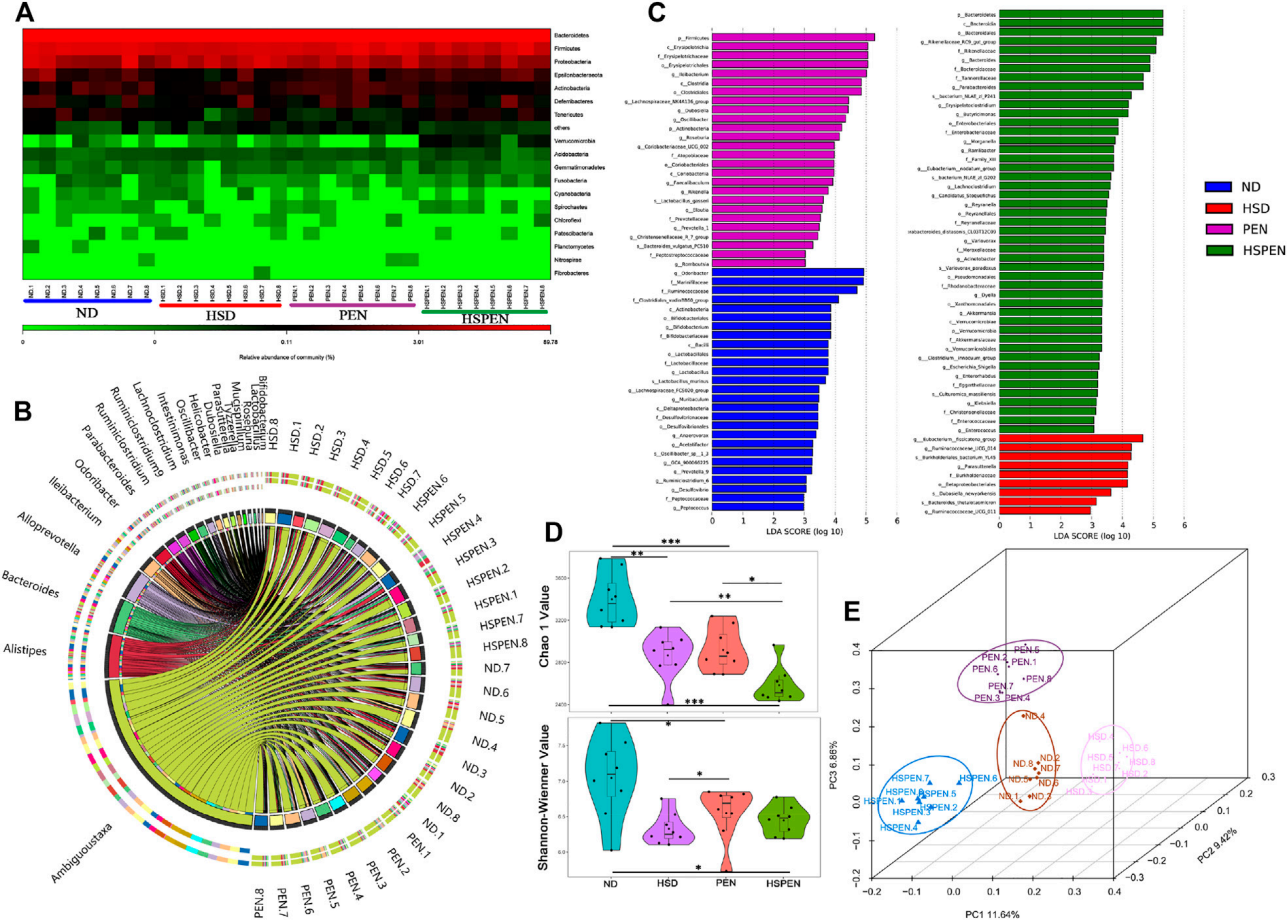
HSD and antibiotic exposure induce dysregulation of the gut microbiome. **(A)** Heatmap showing normalised values of 18 phyla in the faeces of ND-, HSD-, PEN- and HSPEN-treated mice. The normalised abundance values are indicated from red to green, where red indicates that the abundance is the highest and green indicates that it is the lowest. **(B)** The chord diagram reveals the top 20 abundant genera (abundance > 0.2%) in the four groups. **(C)** Histogram scores from linear discriminant analysis (LDA) comparing inter-group variance level by LDA effect size (LEfSe) analysis of the relative abundance. **(D)** Microbial richness estimates (Chao 1 index) and diversity indices (Shannon−Wiener) in the different groups at the 8-weeks point. **(E)** Separation of the faecal bacterial structure expressed using PCoA in the four groups. Data are shown as the mean ± SD; *n* = 8. *p < 0.05, **p < 0.01 and ***p < 0.001.

After removing unqualified sequences, the ND, HSD, PEN and HSPEN groups returned more than 46,329, 63,888, 58,019 and 51,919 effective tags, respectively. The species were annotated using typical OTU 16S rRNA gene sequences. The valid sequences of all OTU-qualified samples were clustered based on ≥97% sequence identity. The heatmap (Figure 4A) shows the normalized values of 18 phyla in the different groups. The mouse intestinal bacteria mainly consisted of Bacteroidetes, with the abundances of 69.16, 74.40, 72.61, and 81.46%, and Firmicutes, with abundances of 19.41, 14.12, 18.54, and 13.13% in the ND, HSD, PEN and HSPEN groups, respectively. The chord diagram reveals the top 20 abundant genera in the faeces (Figure 4B), showing that the three dominant taxa in all groups were *Ambiguous_taxa*, *Alistipes* and *Bacteroides,* accounting for 1.01–39.95% of the entire OTUs. The differential abundance of bacterial genera in the four groups was identified by linear discriminant analysis effect size (LEfSe; Figure 4C). At the genus level, *Odoribacter*, *Bifidobacterium*, *Lactobacillus*, *Muribaculum* and *Anaerovorax*, as biomarkers, were more abundant in the ND group, while *Bateroides*, *Parabacteroides*, *Morganella*, *Ramlibacter*, *Butyricimonas* and *Akkermansia*, as biomarkers, were more abundant in the HSPEN group. The α-diversity—which consists of richness estimates (i.e., Chao 1 index) and diversity values (including Shannon-Wienner indices)—of the microbial communities was measured. The richness estimates of the microbial communities in the HSD, PEN, and HSPEN groups were strikingly decreased compared to the ND group (*p* < 0.01), while the diversity values of the microbial communities in the PEN and HSPEN groups were less than the ND group (*p* < 0.05; Figure 4D). PCoA showed the bacterial diversity among the four groups; 27.92% of the population variance was attributed to the three principal components (PC1, PC2, and PC3), which were consistent and reliable. Overall, the differential abundance among the four groups indicated that high salt intake, antibiotic exposure, and the combination of the two can cause changes in the levels of liver injury and the structure of the mouse gut microbiome.

### Effects of HSD and Antibiotic Exposure on Metabolic Functionality in Mice

**FIGURE 5:**
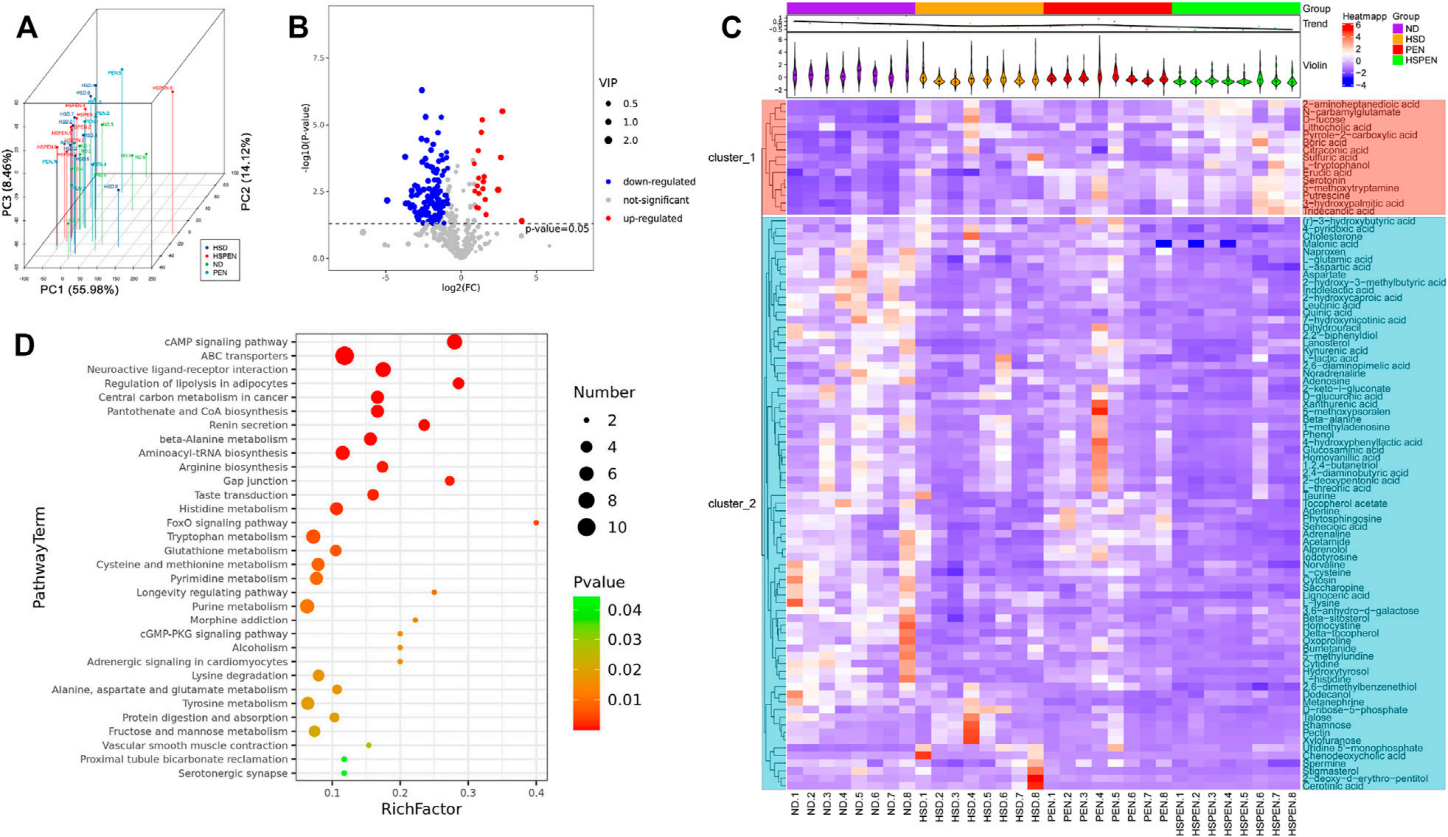
Faecal metabolic analysis in the ND, HSD, PEN, and HSPEN groups. **(A)** Scatterplot of PCoA scores in the various groups. **(B)** The differential variables between the ND and HSPEN groups are shown *via* a volcano plot. Each metabolite is indicated by a dot, with down-regulation represented by red dots, up-regulation represented by blue dots, and no statistical difference represented by green dots. **(C)** Heatmap of 90 metabolites that were differentially (*p* < 0.01) abundant at standardised levels between the ND and HSPEN groups. The distances of the metabolites are expressed by the dendrogram according to their relative abundances. The normalised abundance values are described intuitively from red to blue, expressing the maximum and minimum abundances, respectively. **(D)** Enriched KEGG pathways in the ND group in contrast to the HSPEN group. The statistical significance values (*p* < 0.05) are described intuitively from red to green, showing the most and least differences, respectively. The size of the dot on the vertical axis indicates the metabolite count in the metabolic pathway.

The intestinal microbiota has an effect on the host-microbe metabolic axis, and faecal metabolomics is a helpful tool to detect the interplay between bacteria and host phenotypes (Zierer et al., 2018). The interquartile range denoising method was applied to identify a total of 583 peaks and 468 metabolites that persisted after eliminating background noise. Half of the minimum value was used to replace missing values in the original data. The principal component scores were determined using PCoA (Figure 5A). The results showed that faecal samples clustered significantly, indicating that the faecal metabolites of the four groups were different. PC1, PC2 and PC3 accounted for 55.98, 14.12, and 8.46% of the total variance, respectively, which suggests that the model was consistent and reliable. The volcano plot (Figure 5B) represents the variables with different contents between the ND and HSPEN groups, with each dot representing a metabolite: upregulated metabolites are represented by red dots, downregulated metabolites are represented by blue dots, and metabolites with nonsignificant differences are represented by green dots (*p* > 0.05). However, the volcano plot is complex due to the inclusion of numerous variables. Therefore, the variable importance projection (VIP) value (>1.0) of the orthogonal partial least squares discriminant analysis and the *p* value (<0.01) of a Student’s *t*-test were used to identify 90 differential metabolites between the ND and HSPEN groups. Then, heatmap visualization (Figure 5C) was applied to indicate discrepant metabolites. Totally, 74 metabolites, such as _L_-lactic acid, δ-tocopherol, chenodeoxycholic acid and taurine showed significantly reduced abundance in the faeces of HSPEN-treated mice, while 16 metabolites, including lithocholic acid, erucic acid, putrescine and 3-hydroxypalmitic acid, showed markedly increased abundance as compared to the faeces levels of ND-treated mice (Figure 5C and Supplementary Table S1).

A critical element in the adjustment of tryptophan metabolism to indole derivatives is the intestinal microbiota, and indole derivatives are increasingly recognized as protective molecules against liver disease (Agus et al., 2021). In the present research, in the faeces of HSPEN-treated mice, the level of tryptophan was significantly increased, whereas the levels of indole derivatives—such as indole-lactic acid, indole-3-acetate and 5-methoxyindoleacetate—were significantly reduced compared with the ND-treated mouse faeces (Figure 5C and Supplementary Table S1). The biochemical pathways in the KEGG database were then analysed in relation to the prominent metabolites that differed between the ND- and HSPEN-treated mice. The differentially enriched metabolites in the HSPEN group were all associated with the cAMP signalling pathway, ABC transporters, neuroactive ligand−receptor interaction and regulation of lipolysis in adipocytes [*p* < 0.01, frequency-distance-relationship (FDR) correction; Figure 5D].

### Integration of Microbiomic and Metabolomic Analyses

**FIGURE 6:**
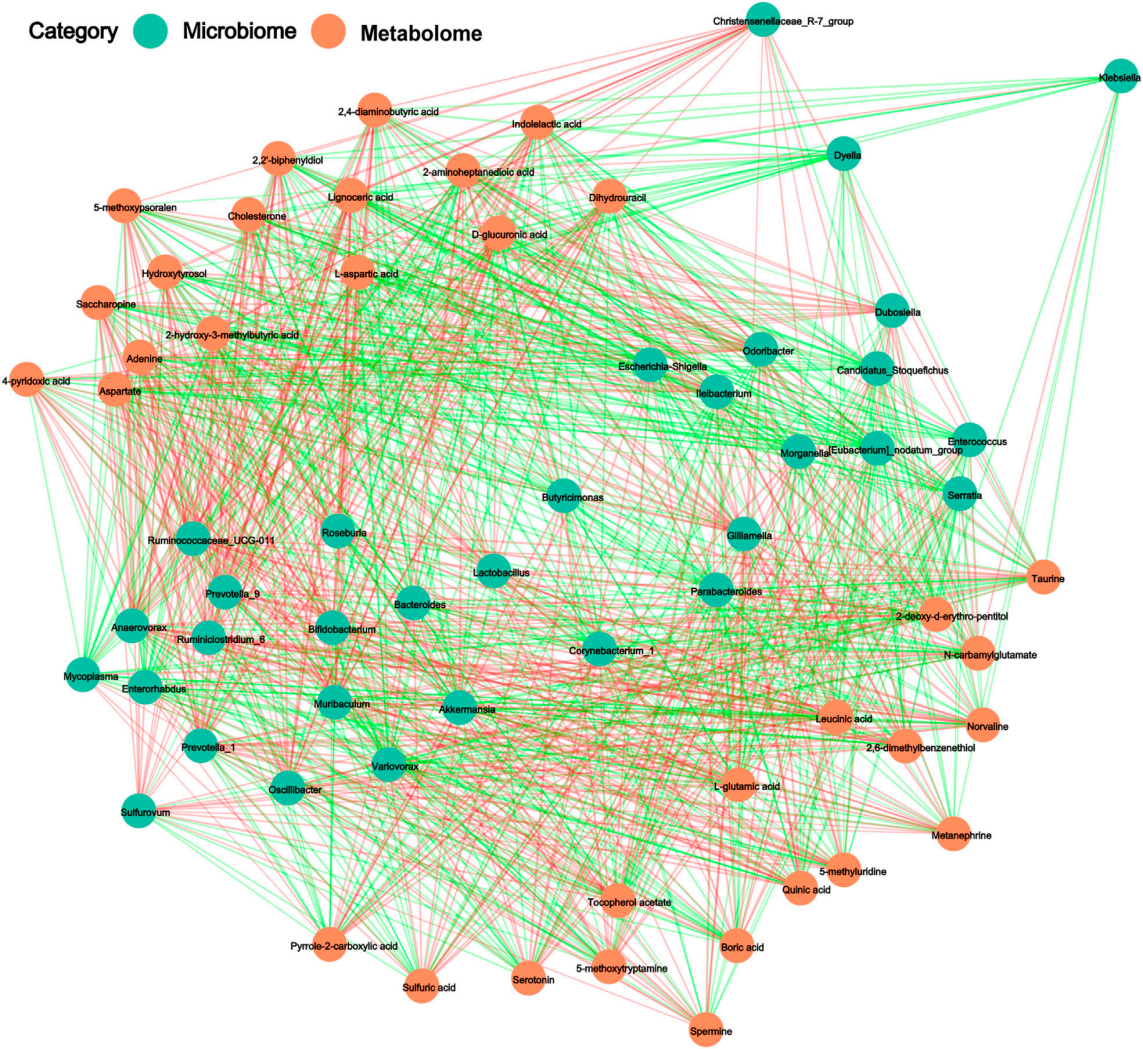
The regulatory networks between 32 gut microbiota (*p* < 0.01) and 33 faecal metabolites (*p* < 0.001) in the ND group in comparison with the HSPEN group. The Pearson’s rank correlations (R-value > 0.40) are expressed as red and green lines, representing positive and negative correlations, respectively.

The correlations between the intestinal microbiota and their metabolites were determined using Pearson’s correlation analysis. Positive and negative correlations between the determined genera and the abundances of metabolites are presented through the resulting metabolic association networks (Figure 6). Supplementary Table S2 shows the R-values for these associations. The predominant genera in the ND group, including *Odoribacter*, *Bifidobacterium*, *Lactobacillus*, *Muribaculum*, and *Anaerovorax*, displayed strong positive correlations with taurine, tocopherol acetate, indole-lactic acid and 4-pyridoxic acid. The dominant genera in the HSPEN group, including *Bateroides*, *Parabacteroides*, *Morganella*, *Butyricimonas*, and *Akkermansia*, displayed strong positive correlations with 2-aminoheptanedioic acid, N-carbamylglutamate, 5-methoxytryptamine, boric acid, sulfuric acid and pyrrole-2-carboxylic acid. Collectively, high salt intake, antibiotic exposure and the combination of the two can influence bacteria components and significantly alter the faecal metabolome.

### HSD and Antibiotic Exposure Affect the Gut Function and Morphology in Mice

**FIGURE 7:**
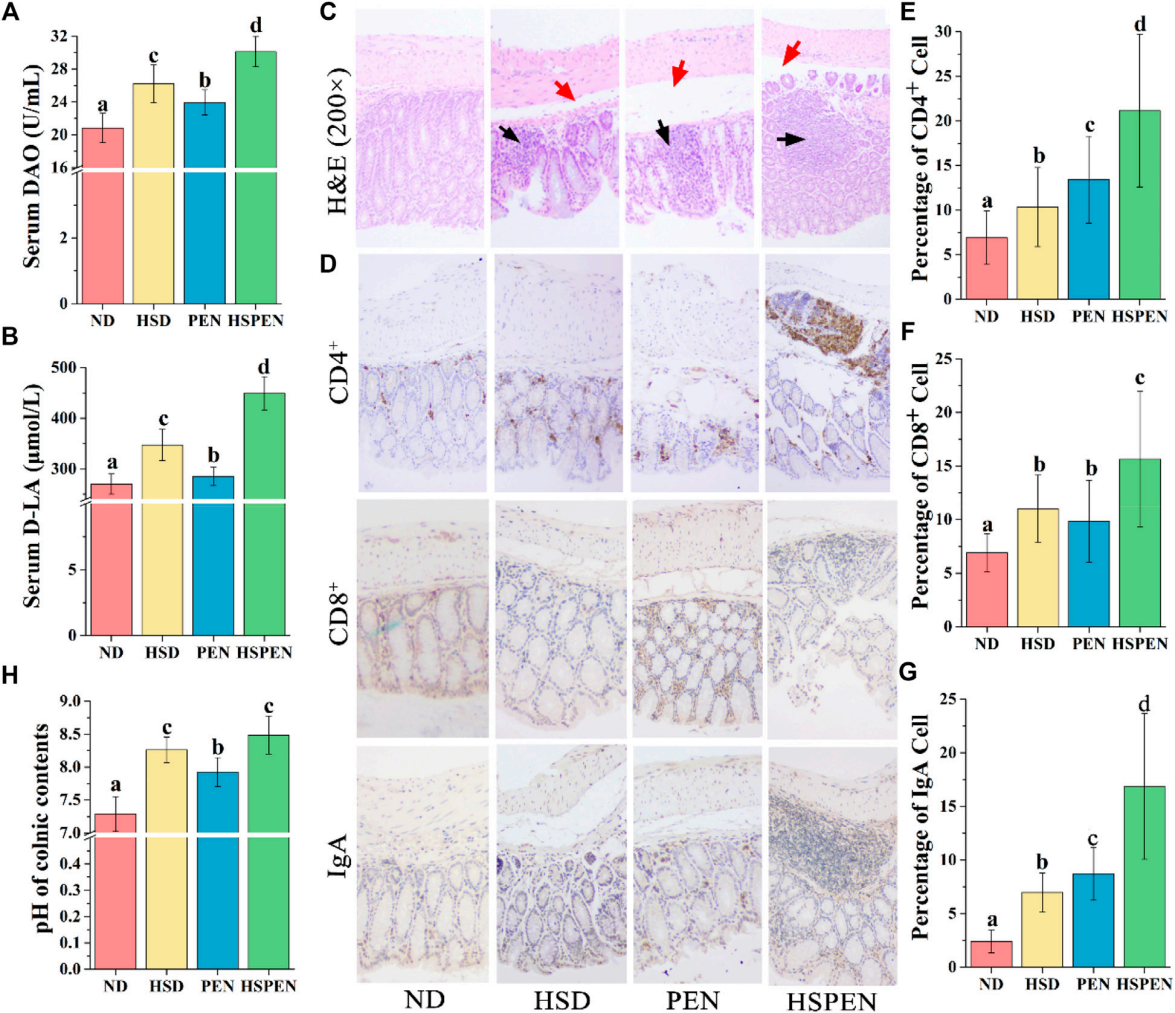
Effects of HSD and antibiotic exposure on colonic function and morphology in mice. Levels of DAO **(A)** and D-LA **(B)** in serum, which signify the intestinal permeability of the ND-, HSD-, PEN- and HSPEN-treated mice. **(C)** Photomicrographs (×200 magnification) of the colon in the various groups of mice. Inflammatory cell osmosis and oedema in the submucosal layer are expressed respectively by black and red arrows. **(D)** Microscopic observation of CD4^+^, CD8^+^, and IgA lymphocytes on the colon epithelium (×200 magnification). Image Pro-Plus 6.0 software was employed to analyse the average numbers of CD4^+^ **(E)**, CD8^+^ **(F)**, and IgA **(G)** lymphocytes in the colon tissues and the **(H)** pH values of the colonic constituents of the groups. Data are expressed as the mean ± SD; *n* = 8. Different letters were significantly different (*p* < 0.05).

The tissues and function of the colon are affected by changes in the constitutes of the gut flora, since the colon contains 90% of the microorganisms in the host (Savage, 1977). Thus, the intestinal permeability, immunologic function of colonic epithelial cells, and pathology of the ND, HFD, PEN, and HSPEN groups were observed. The influences of HSD and antibiotic exposure on intestinal permeability in mice were investigated by measuring DAO and _D_-LA levels in the serum. DAO activity in serum and the concentration of _D_-LA were dramatically increased (*p* < 0.05) in the ND group compared to the HSD, PEN, and HSPEN groups, indicating that intestinal permeability in the HSD-, PEN- and HSPEN-treated mice was higher (Figures 7A,B). In particular, the permeability indices in the HSPEN group were elevated compared with those in the HSD and PEN groups (*p* < 0.05; Figures 7A,B). The results of IHC staining for intestinal immune responses (×200 magnification) revealed the expressions of CD4^+^, CD8^+^ and IgA in colonic epithelial cells and mucosal lymphocytes (Figure 7D). CD4^+^ (Figure 7E), CD8^+^ (Figure 7F) and IgA (Figure 7G) protein expressions in the HSPEN group were significantly elevated compared with those in the other three groups (*p* < 0.05). The structures of the mucosal, submucosal, muscularis, and serous layers of the colonic tissues in the ND group were distinct and the borders were obvious. However, in the HSD, PEN, and HSPEN groups, there was submucosal oedema and space enlargement (red arrows), as well as visible inflammatory cell infiltration between the mucosal glands (black arrows) (Figure 7C). Moreover, the pH of the colonic contents was dramatically higher in the HSD and HSPEN groups than the ND and PEN groups (*p* < 0.05); there was no obvious difference in pH between the HSD and HSPEN group (*p* > 0.05; Figure 7H).

## Discussion

Poor health and even various diseases can be caused by long-term antibiotic exposure and high salt consumption. Epidemiological evidence suggests an association between these two risk factors in the process of NAFLD (Choi et al., 2016; Schneider et al., 2021). Several studies have demonstrated the exacerbation of liver damage in relation to the combination of two or multiple risk factors, such as drug abuse, ambient fine particulate matter, and unhealthy dietary habits (Mahana et al., 2016; He et al., 2022). Our previous study confirmed that both HSD and alcohol consumption strongly influence microbial composition, and consumption of a HSD with alcohol promotes the process of fatty liver disease development (Chen et al., 2020a). Uetake et al. (2015) found that HSD for 8-weeks could cause inflammation and fibrosis in liver steatosis induced by oxidative stress and dyslipidemia in mice. Mahana et al. (2016) demonstrated that penicillin G exposure for 8-weeks significantly increased the body fat and insulin resistance, and penicillin G plus dietary fat exacerbated obesity, type 2 diabetes and NAFLD due to microbiome dysbiosis in mice. As expected, a HSD or antibiotics (specifically, penicillin G) alone produced liver steatosis and injury in this study, while their combination exacerbated the progression toward liver damage (Figure 2E and Table 1). However, evidence to date on the effects of antibiotics on liver function has been conflicting. For instance, Bergheim et al. (2008) indicated that fructose-induced hepatic lipid accumulation and endotoxemia can be markedly reduced by concomitant treatment with antibiotics (e.g., neomycin) for 8 weeks in mice. The opposite effects of antibiotics on liver function have contributed to confusion regarding the clinical application of antibiotics. The potential mechanism underlying the opposite effects of antibiotics on liver function may involve the category of antibiotics administered to the host. Firstly, penicillin G, as a β-lactam antibiotic, can cause gut bacteria to release significant quantities of peptidoglycan subunits which potently induce a peptidoglycan storm, resulting in NAFLD in mice (Cho et al., 2014; Ji et al., 2019; Tan et al., 2021). In contrast, aminoglycosides, such as neomycin, can disorganize the bacterial cell envelope, as manifested by major perturbation in peptidoglycan and lipopolysaccharide biosynthesis (Han et al., 2019; Hussein et al., 2020). Further, antibiotic classes display obvious inhibition spectra and behaviours, including phylogeny independence for β-lactams, which can strongly influence the effect of the antibiotics on the host’s gut microbiome (Maier et al., 2021).

Blood from the intestine flows through the portal venous system to the liver, comprising more than two-thirds of the hepatic blood; thus, there is a close link between the intestine and liver, the so-called “gut–liver” axis. The blood flow drives several microbial derivates to the liver, including harmful and beneficial metabolites (Ianiro et al., 2016). Increasing evidence indicates that intestinal flora plays a pivotal role in the pathogenesis of several chronic liver diseases (Tripathi et al., 2018). As previously observed (Mahana et al., 2016; Zhang et al., 2019b), in the current study, HSD and penicillin G changed the microbial community composition in mice, with structural differences between the four groups. Here, a HSD with or without penicillin G significantly lowered the abundance of probiotics (such as *Bifidobacterium* and *Lactobacillus*) in the gastrointestinal tract compared to the control animals (Figure 4C). Strikingly, in this study, the microbiota of the HSPEN group was extremely abundant with *Akkermansia* species (Figure 4C). Previous studies have indicated that *Akkermansia* may be regulated by bile acids and increased levels of bile acids can reduce the population of *Akkermansia* in mice (Higarza et al., 2021; Keshavarz Azizi Raftar et al., 2021). This finding seems to be in contradiction with the acknowledged enrichment of probiotic species within this genus. Interestingly, increased abundances of faecal *Akkermansia* has been found in individuals with a high-fat high-sucrose diet (Carmody et al., 2015) and patients with NAFLD (Moreira et al., 2018), inflammatory bowel disease (Seregin et al., 2017), acute graft-versus-host disease (Yang et al., 2021), and multiple sclerosis (Berer et al., 2017; Cekanaviciute et al., 2017). Thus, these results suggest that intervention with probiotics (especially *Akkermansia*) in patients with a HSD or/and penicillin G-associated health problems should be applied with caution. In this study, the HSD, antibiotics, and the combination of the two resulted in an overall decrease in intestinal microbial diversity (Figure 4D). This is consistent with the findings of Moghadamrad et al. (2019), indicating that complicated gut microbiota may play a hepatoprotective role.

Metabolism of tryptophan to indoles can be efficiently accomplished by the intestinal microbiota, thereby reducing inflammation and the fatty degeneration of the liver. This study verified that the abundance of tryptophan in the faeces of HSD-, PEN- and HSPEN-treated mice was markedly elevated compared to ND-fed mice (Figure 5C and Supplementary Table S1). In particular, intestinal permeability, inflammatory responses and the accumulation of ROS in the intestine can be reduced by indole-lactic acid, indole-3-acetate and 5-methoxyindoleacetate, as endogenous and beneficial metabolites (Wlodarska et al., 2017; Ehrlich et al., 2018). Furthermore, 3-hydroxypalmitic acid can significantly induce mitochondrial dysfunction to contribute to the severe hepatic clinical manifestations observed in affected patients (Cecatto et al., 2018). Putrescine and erucic acid can induce lipid dysmetabolism and intrahepatic cholestasis in animal models (Shelepov et al., 1990; Reyes et al., 1995). In the present study, the abundances of 3-hydroxypalmitic acid, putrescine, and erucic acid were dramatically increased in the HSPEN group, while the abundances of beneficial metabolites from the gut microbiota, including _L_-lactic acid (Shan et al., 2020), δ-tocopherol (Bril et al., 2019) and chenodeoxycholic acid (Malhi and Camilleri, 2017), were significantly decreased in comparison with the ND group (Figure 5C and Supplementary Table S1); these changes may induce harmful effects on the liver function. Moreover, metabolites associated with the cAMP signalling pathway, ABC transporters, neuroactive ligand−receptor interaction and regulation of lipolysis in adipocytes were dramatically changed in the HSPEN group compared with the ND group (Figure 5D); these pathways are involved in cystic fibrosis, obesity and NAFLD (Reynisdottir et al., 1995; Manson et al., 2011; Fuchs et al., 2014). Increased intestinal permeability is the main cause of the inflammatory reactions and immune responses that induce hepatic disorders (Chopyk and Grakoui, 2020). Penicillin is only bactericidal when it is applied at the early growth stages; however, as the bacterial composition changes, susceptibility decreases (Moore et al., 2003). Previous studies have indicated enhancement of pro-inflammatory activity by penicillin during treatment for inflammatory diseases (Stevens et al., 1993; Moore et al., 2003). In this study, the IgA reaction and T cell (e.g., CD4^+^) release in the PEN group were significantly increased compared to the HSD and ND groups (Figures 7E,G), indicating higher inflammatory reactions caused by antibiotic exposure in the intestine. The limitless movement of toxic metabolites and pro-inflammatory cytokines through the damaged colonic barrier (Figures 7A,B) into the portal venous system can induce hepatotoxicity *in vivo*.

Mitochondrial dysfunction is reported to be involved in the progression of steatohepatitis (Loomba et al., 2021). Recently, evidences have confirmed that mitochondrial function is related to the intestinal microbiota, which may be linked with microbial metabolites (Begriche et al., 2006; Zhang et al., 2021). The results of this study revealed impairment in mitochondrial integrity (Figure 3A) and microbial metabolic function (Figure 3B) in the HSD and PEN groups, especially in the HSPEN group; this is consistent with the pathological findings of liver damage (Figure 2E). To further confirm the molecular mechanism by which salt consumption, antibiotic exposure and their combination caused mitochondrial abnormalities in hepatocytes, ROS accumulation and H_2_O_2_ release were detected in the current study. Consistent with previous studies (Chen et al., 2020b; Zhang et al., 2021), substantial ROS accumulation in hepatocytes induced lipid dysmetabolism and liver damage in HSPEN-treated mice. Mitochondrial dysfunction induces accumulation of fatty acids, which are partly metabolized by peroxisomes (Fritz et al., 2007) and microsomes, and this is followed by ROS production and lipid peroxidation. Moreover, products such as MDA (Table 1) have a longer half-life than ROS and can spread to other areas of the body to induce oxidative stress (Rolo et al., 2012; Borrelli et al., 2018). Together, all findings suggest that mitochondrial and liver function may be damaged under HSD and antibiotic exposure. Overall, long-term salt and antibiotic exposure changed the structure of the gut microbiota and its metabolites, aggravated intestinal dysfunction, caused hepatic mitochondrial abnormalities, induced hepatic lipid accumulation, and contributed to NAFLD progression.

## Conclusion and Perspectives

To the best of our knowledge, this is the first study to present a global view of the combined effects of sodium and antibiotic exposure on the liver health of mice. The results of this study indicated that these two risk factors might affect hepatic functions and induce gut microbial dysbiosis and metabolic disorders. Antibiotic exposure may enhance the intestinal immune response and induce ROS accumulation and mitochondrial abnormalities in hepatocytes, thereby aggravating the adverse effects observed in HSD-fed mice. Due to the popularity of antibiotic abuse and exposure worldwide, the findings of this study not only contribute to a better understanding of the molecular mechanism underlying the hepatic response to antibiotic exposure and the excessive intake of salt/sodium but also provide a model for studying the toxicological mechanism of antibiotics in multi-organ systems. However, the mouse’s average daily salt consumption was significantly higher than their tolerance in this study, which likely does not occur in humans for extended periods (Relman, 2017). Human hepatic injury results from excessive salt intake and other clinical and environmental factors over long periods (Meola et al., 2016). Thus, the effects of HSD enhanced antibiotic-induced hepatic injury and mitochondrial abnormalities requires further clinical and epidemiological investigation.

## Data Availability

The datasets presented in this study can be found in online repositories. The names of the repository/repositories and accession number(s) can be found below: The raw sequencing data have been uploaded in the Sequence Read Archive (SRA) of NBCI (accession number: PRJNA665282).
